# Transparent qualitative model-building in high-stakes digital governance: a methods protocol for tracing institutional mechanisms

**DOI:** 10.3389/frma.2026.1889262

**Published:** 2026-07-27

**Authors:** Ahmad Nur Hidayat, Nina Karlina, Ramadhan Pancasilawan, R. Widya Setiabudi Sumadinata

**Affiliations:** Department of Public Administration, Faculty of Social and Political Sciences, Universitas Padjadjaran, Bandung, Indonesia

**Keywords:** digital governance, electoral management bodies, embedded case study, qualitative research methods, research transparency

## Abstract

Digital governance research often identifies what digital systems do but less frequently explains how institutions make those systems governable under legal, organizational, political, and public scrutiny. This methodological problem is especially consequential in election administration, where digital records and platforms affect voting rights, political eligibility, logistics, public information, and the credibility of results. Existing technical audits, adoption studies, maturity assessments, usability evaluations, and trust surveys provide important evidence, but they rarely make the institutional route from records and actor accounts to mechanism-based explanation fully inspectable. This article develops a critical realist qualitative methods protocol for transparent institutional mechanism tracing in high-stakes digital governance. The protocol is not a general framework for all qualitative data analysis; it is specifically designed to reconstruct how organizational capacities are translated into validation, correction, coordination, escalation, auditability, explanation, accountability, and institutional learning. An electoral management body is treated as the main case, while voter data, party and candidate verification, logistics, and results publication constitute embedded analytical arenas. The operational workflow integrates purposive and functionally stratified sampling, process-oriented interviews, document mapping, abductive and mechanism-oriented coding, cross-arena matrices, evidence-strength decisions, rival-explanation assessment, negative-case analysis, audit trails, expert validation, positionality documentation, and tiered transparency. A bounded pilot vignette using publicly available documents from Indonesia's 2024 electoral administration demonstrates how regulatory evidence and institutional explanations can be transformed into action codes, matrix entries, and an evidence-graded candidate mechanism. The protocol contributes an operationally explicit, reviewable, and ethically safeguarded route from qualitative evidence to institutional explanation.

## 1 Introduction

Qualitative research is essential for studying institutions whose work cannot be reduced to indicators, interfaces, or formal rules. Case study and qualitative inquiry are especially useful for reconstructing how institutional actors interpret mandates, how records move across organizational units, how errors are corrected, and how responsibility is assigned when decisions become contested (Yin, 2018; Levitt et al., 2018; Maxwell, 2012). Yet qualitative studies of complex institutions face a recurring problem: the route from empirical material to explanatory model is often difficult to inspect. Readers may see rich description, thematic categories, or a final diagram, but not the analytical steps through which interviews, documents, observations, and public records become mechanism-based claims (Nowell et al., 2017; Aguinis and Solarino, 2019; Bingham, 2023). The issue is therefore not simply how to collect qualitative evidence, but how to make the movement from evidence to coding, from coding to comparison, and from comparison to model-building transparent enough for review, adaptation, and cumulative research (Elman et al., 2018; Moravcsik, 2014; Timmermans and Tavory, 2012).

Existing qualitative studies of complex institutions can provide rich descriptions, thematic categories, and final models, but the analytical route from evidence to explanation often remains difficult to inspect (Nowell et al., 2017; Aguinis and Solarino, 2019; Bingham, 2023). The problem is not a lack of qualitative traditions; grounded theory, discourse analysis, institutional ethnography, narrative inquiry, content analysis, and interpretive case analysis already offer established approaches (Strauss and Corbin, 1998; Charmaz, 2014; Fairclough, 1995, 2003; Smith, 2005; Krippendorff, 2019). The unresolved problem is how researchers can transparently reconstruct institutional mechanisms from interviews, documents, observations, and public records when digital systems operate under legal, organizational, political, and security constraints. Existing evaluations commonly emphasize technical audits, adoption, maturity, usability, service performance, or public trust, while the institutional work of validating records, authorizing corrections, assigning responsibility, documenting decisions, explaining outputs, and learning from failures remains insufficiently traced (Janssen et al., 2012; Norris, 2011; Garnett and James, 2020; Busuioc, 2021). This gap is especially consequential in electoral administration, where digital systems affect voting rights, political competition, logistics, and the credibility of results. Without an inspectable route from corpus construction to coding, comparison, rival-explanation assessment, evidence-strength judgment, and model refinement, qualitative models risk appearing as interpretive assertions rather than reviewable explanations.

The purpose of this study is to develop and demonstrate a critical realist qualitative methods protocol for transparent, reviewable, and ethically safe institutional mechanism tracing in high-stakes digital governance. The study is deliberately bounded: it does not offer a comprehensive framework for all qualitative data analysis traditions, nor does it conduct a cybersecurity audit, software evaluation, or population-level assessment of trust. Instead, it treats an electoral management body as the main case and four digital arenas—voter data, party and candidate verification, logistics, and results publication—as embedded units through which institutional work can be reconstructed. The protocol specifies how researchers can build an evidentiary corpus, conduct process-oriented interviews and document analysis, apply abductive and mechanism-oriented coding, compare arenas through matrices, assess rival explanations and evidence strength, preserve audit trails, validate emerging claims, and use tiered transparency to protect sensitive information. Electoral administration is used as a demanding methodological setting because its digital outputs are produced under compressed timelines, legal constraint, partisan scrutiny, and strong expectations of accuracy, auditability, accountability, and public explanation (Birch, 2011; Norris, 2014; James et al., 2019; Garnett, 2019).

Accordingly, the study has four objectives. First, it seeks to identify the institutional work through which public organizations make digital systems governable, including validation, correction, coordination, escalation, documentation, explanation, accountability, and learning. Second, it aims to trace how organizational capacities—strategic, structural-coordinative, human resource, technological-data, communication-legitimacy, and evaluation-learning capacities—are translated into observable governance practices and institutional conditions. Third, it establishes a documented analytical route from sensitizing concepts to codes, cross-arena matrices, mechanism statements, evidence-strength labels, negative-case analysis, expert validation, and model refinement. Fourth, it defines practical standards for transparency, confidentiality, ethical redaction, and analytical transferability in politically sensitive and data-intensive institutional settings. Together, these objectives are intended to produce a mechanism-based qualitative model that is empirically grounded, open to review, bounded by evidence, and adaptable to other electoral management bodies and high-stakes public institutions.

The protocol is grounded in a critical realist understanding of qualitative explanation. It assumes that institutional mechanisms are real in their effects but not always directly observable in their entirety. Researchers therefore infer mechanisms from patterned evidence, documented routines, actor accounts, institutional texts, observed practices, contradictions, and absences. This position differs from a purely interpretivist account that would treat mechanisms mainly as meanings constructed by actors, and it also differs from positivist designs that require standardized measurement before explanation can begin. In this protocol, validity rests on whether a mechanism claim is plausible, evidence-supported, rival-aware, bounded by stated conditions, and open to revision when negative cases or missing evidence weaken the explanation.

The article develops this logic into an embedded qualitative model-building protocol. An electoral management body is treated as the main case, while four digital arenas—voter data, party and candidate verification, logistics, and results publication—serve as embedded units of analysis. This design prevents two common errors: treating the institution as a single undifferentiated actor, or treating each platform as a self-contained technical artifact. The protocol moves from case and embedded-unit selection to corpus construction, document mapping, pilot interviews, abductive coding, mechanism-oriented coding, cross-arena matrices, pattern matching, explanation building, negative case analysis, expert validation, redaction logging, and model refinement. Coding is treated as an intermediate analytical step, not as the result itself. The expected product is a model showing how capacities such as coordination, data control, legal interpretation, communication, and learning are translated into validation, correction, auditability, escalation, explanation, accountability, and procedural revision.

Several methodological traditions inform the protocol. Case study research supports the analysis of institutional processes embedded in real-life settings (Yin, 2018). Abductive analysis allows researchers to begin with sensitizing concepts while remaining open to evidence that revises them (Timmermans and Tavory, 2012). Process tracing, pattern matching, and explanation building help move analysis from description toward mechanism-based claims (Fairfield and Charman, 2017; Ricks and Liu, 2018; Beach and Pedersen, 2019). Qualitative transparency literature further emphasizes audit trails, data management, reflexive memoing, and documented analytical decisions (Nowell et al., 2017; Levitt et al., 2018; Bingham, 2023).

The contribution is methodological rather than evaluative. The article does not provide a cybersecurity audit, software evaluation, or general theory of qualitative data analysis. It offers a transparent protocol for mechanism-oriented qualitative model-building in high-stakes digital governance. The protocol contributes by converting embedded case study design into a stepwise workflow; identifying inspectable artifacts such as corpus registers, codebooks, cross-arena matrices, mechanism statements, validation memos, redaction logs, and evidence-strength tables; clarifying how transparency can be pursued without unsafe disclosure; and positioning digital electoral governance as a demanding setting for studying data-intensive public institutions whose outputs affect rights, legitimacy, accountability, and public defensibility.

## 2 Materials and equipment

### 2.1 Purpose and readiness logic

In this protocol, materials and equipment are not laboratory supplies but the evidentiary, analytical, and protective resources needed to make qualitative model-building inspectable. The study requires no reagents, technical testing devices, or penetration tools. It requires an infrastructure for showing how evidence is gathered, classified, coded, compared, interpreted, validated, redacted, and reported. Four functions guide the selection of materials: building a credible corpus, preserving an audit trail, protecting participants and sensitive information, and making the path from evidence to model reviewable. This emphasis is consistent with qualitative case study design, reporting standards, and work on research transparency (Yin, 2018; Levitt et al., 2018; Bingham, 2023; Fradkin and Mugnaini, 2021; Ferreira et al., 2026).

### 2.2 Evidentiary corpus

The evidentiary corpus consists of interviews, documents, and, where feasible, observation records. These materials are selected not to produce statistical representativeness, but to reconstruct institutional action sequences across different segments of the digital governance chain (Yin, 2018; Patton, 2015; Maxwell, 2012). The central sampling question is therefore not “how many respondents represent the population?,” but “which information sources are necessary to trace how records are validated, corrected, authorized, explained, contested, and made auditable?” This logic is consistent with purposive and criterion-based qualitative sampling, where participants and materials are selected for their informational relevance rather than numerical representativeness (Patton, 2015; Campbell et al., 2020; Malterud et al., 2016).

The interview corpus should include participants who can reconstruct different segments of the governance chain. At minimum, it should cover institutional leaders, legal or procedural officers, data and technology staff, regional or local implementers, system operators, oversight or dispute actors, civil society or electoral observers, affected users, and independent experts. This diversity reduces elite bias, official-account bias, and platform bias by preventing mechanism claims from relying on one actor category, one institutional level, or official narratives alone (Aguinis and Solarino, 2019; Patton, 2015; Campbell et al., 2020). It also supports information power by prioritizing participants who hold direct knowledge of the institutional process being reconstructed (Malterud et al., 2016).

Semi-structured interviews should be organized around process reconstruction rather than general opinion. Questions should ask how records are checked, who authorizes correction, where decisions are documented, how problems are escalated, how digital outputs are explained, and what evidence remains for later review. This approach preserves comparability across participant categories while allowing probes into context-specific routines, documents, decisions, and meanings (Kallio et al., 2016; Patton, 2015). It also aligns interview data with mechanism tracing and reduces the risk that the analysis remains at the level of broad perceptions or abstract themes (Timmermans and Tavory, 2012; Beach and Pedersen, 2019; Ricks and Liu, 2018).

The document corpus includes legal and procedural instruments, technical and operational guidelines, data protection and cybersecurity policies, public statements, oversight reports, complaint records, and dispute materials. Document analysis is central because formal texts reveal mandates, procedural boundaries, institutional claims, evidentiary hierarchies, and changes over time (Bowen, 2009). Each item should be logged by title, issuer, date, legal status, arena, access condition, sensitivity level, and evidentiary value. Documents should also be used to anchor and check interview accounts, because legal rules, internal manuals, public statements, complaint records, and oversight reports do not carry the same authority, audience, or proximity to practice (Bowen, 2009; Yin, 2018; Prior, 2008).

Observation records are optional but valuable when access is permitted because they allow researchers to document visible institutional practice rather than relying only on retrospective accounts or formal documents (Patton, 2015; Yin, 2018). Observation should focus on role allocation, document use, rule references, escalation routines, correction discussions, coordination, and authority clarification. Researchers should not record passwords, protected personal data, system architecture, security configurations, vulnerabilities, or operationally sensitive details because confidentiality and harm avoidance are central to ethical qualitative research in sensitive institutional settings (Kaiser, 2009; Saunders et al., 2015). When observation is not possible, the limitation should be logged and compensated through interviews, documents, public records, and expert validation.

Together, interviews, documents, and observation records allow the researcher to distinguish between formal mandate, reported practice, visible practice, and contested practice. A mechanism claim should become stronger only when source types converge, rival explanations have been considered, and evidence supports the proposed action sequence (Denzin, 1978; Lincoln and Guba, 1985; Nowell et al., 2017; Beach and Pedersen, 2019). If one source type is unavailable, the researcher should record the limitation and adjust the evidence-strength label rather than presenting restricted or incomplete evidence as fully supported (Levitt et al., 2018; Aguinis and Solarino, 2019).

### 2.3 Field instruments

Six instruments should be prepared before fieldwork: an interview protocol, document review matrix, observation guide, coding guide, cross-arena matrix, and expert validation form. These instruments standardize data collection, classify documentary evidence, guide safe observation, support coding transparency, compare embedded arenas, and assess the plausibility and limits of emerging mechanism claims (Kallio et al., 2016; Bowen, 2009; Yin, 2018; Bingham, 2023; Colson and Cooke, 2018).

### 2.4 Analytical systems and audit trail

Analytical equipment includes QDA software, spreadsheet or matrix software, an audio recorder when permitted, a transcription system, encrypted storage, a version-control log, and a redaction template. QDA software such as NVivo, ATLAS.ti, MAXQDA, Dedoose, or an equivalent tool can support coding, memoing, retrieval, and traceability, but the software should be treated as an analytical aid rather than a substitute for researcher judgment (Silver and Lewins, 2014; Bingham, 2023). Spreadsheet or matrix software is needed for corpus registers, sampling logs, document matrices, cross-arena comparison, and validation records because qualitative analysis often requires systematic displays that make patterns, contrasts, and evidence chains visible (Miles et al., 2014; Yin, 2018). The version-control log should record changes to instruments, code definitions, inclusion decisions, matrix structure, mechanism maps, redaction rules, validation responses, and reporting choices. Such documentation strengthens auditability, transparency, and trustworthiness in qualitative research (Nowell et al., 2017; Levitt et al., 2018). Redaction templates and encrypted storage are also necessary because sensitive qualitative materials require confidentiality protection, anonymization, and careful management of disclosure risk (Kaiser, 2009; Saunders et al., 2015). Without this documentation, reviewers may reasonably read the final model as interpretive assertion rather than traceable analysis (Aguinis and Solarino, 2019). Table 1 summarizes the required materials, instruments, analytical tools, and their methodological functions.

**Table 1:** Materials, instruments, and methodological functions.

Category	Required item	Methodological function
Data materials	Interview transcripts	Reconstruct routines, decisions, constraints, interpretations, and action sequences
Data materials	Legal and procedural documents	Establish mandates, authority, legal status, correction pathways, and boundaries
Data materials	Public statements, oversight records, and observation notes	Trace explanation, contestation, institutional response, and visible practice
Data materials	Validation records	Document expert feedback, uncertainty, model revision, and boundary conditions
Instruments	Interview protocol and document matrix	Standardize questioning and classify documentary evidence
Instruments	Observation and coding guides	Keep observation safe and support consistent coding and comparison
Instruments	Cross-arena matrix and validation form	Compare mechanisms and test plausibility, transferability, and limits
Analytical tools	QDA software, spreadsheets, secure storage, version log, and redaction template	Support coding, retrieval, matrices, data protection, audit trails, and safe reporting

### 2.5 Minimum readiness package

Before data collection begins, researchers should prepare five readiness files: a sampling log, corpus register, instrument packet, data protection plan, and reporting checklist. The sampling log justifies participant categories and embedded arenas; the corpus register records interviews, documents, observations, validation records, memos, and redactions; and the instrument packet standardizes interview, document, observation, and coding procedures (Patton, 2015; Yin, 2018; Kallio et al., 2016). The data protection plan specifies consent, storage, anonymization, redaction, and reporting restrictions, while the reporting checklist identifies which artifacts can be public, supplementary, restricted, or non-disclosable (Kaiser, 2009; Saunders et al., 2015; Levitt et al., 2018). Any fieldwork change should be dated, justified, and preserved in the audit trail (Nowell et al., 2017; Tracy, 2010).

### 2.6 Data security and safe reporting

Data security should be treated as part of method quality because secure data management supports both research integrity and participant protection (Corti et al., 2020; Levitt et al., 2018). Recordings, transcripts, matrices, consent files, and validation records should be stored in encrypted folders or approved institutional systems. Identifying information should be separated from anonymized transcripts and analytical files, and access should be limited to authorized researchers. File names should use participant codes rather than personal names. Audio files should be deleted or archived according to approved ethics procedures after transcription and verification.

Politically sensitive research also requires careful reporting safeguards. Participants may face professional, political, legal, or reputational risk if statements are linked to names, positions, offices, or specific incidents. Confidentiality therefore shapes both ethics and evidence quality (Kaiser, 2009; Saunders et al., 2015). Researchers should prepare anonymization rules, redaction procedures, and criteria for aggregating participant categories before analysis begins because anonymization in qualitative research is not merely technical removal of names, but also involves protecting contextual identities and relational clues (Tolich, 2004; Saunders et al., 2015). Reports should describe governance mechanisms without exposing passwords, protected personal data, system architecture, security configurations, or operational vulnerabilities. Each redaction decision should be logged with its rationale and effect on interpretation so that confidentiality decisions remain transparent and reviewable (Aguinis and Solarino, 2019; Corti et al., 2020). These safeguards allow researchers to report institutional governability without turning transparency into avoidable harm.

## 3 Methods

### 3.1 Design logic and epistemological orientation

This protocol is not intended as a general framework for all qualitative data analysis traditions, nor is it limited to organizing, storing, or retrieving qualitative material. Its specific purpose is to support mechanism-oriented qualitative model-building in studies where digital systems operate as institutional arrangements with legal, organizational, and public consequences. The central analytical question is: through which mechanisms does organizational capacity become digital governance practice? To address this question, the protocol has four operational objectives: to identify the institutional work performed around digital systems; to trace how organizational capacities are translated into validation, correction, coordination, auditability, explanation, accountability, and learning; to document the analytical movement from evidence to codes, matrices, mechanism statements, and model refinement; and to establish standards for reviewability, confidentiality, and transferability in high-stakes institutional research. Coding and data organization are therefore treated as intermediate analytical operations rather than as the final objective of the protocol.

Epistemologically, the protocol is grounded in a critical realist orientation with interpretivist sensitivity to institutional meaning, context, and actor interpretation (Bhaskar, 1978; Sayer, 2000; Maxwell, 2012). It assumes that institutional mechanisms are real in their effects but not directly observable as fixed objects. They must be reconstructed through interviews, documents, observations, public records, and traces of administrative action. This position shapes the protocol in three ways. First, mechanisms are treated as evidence-supported explanatory pathways linking capacity, action, condition, and consequence (Beach and Pedersen, 2019; Fairfield and Charman, 2017). Second, validity is pursued through triangulation, rival-explanation assessment, negative case analysis, reflexive memoing, and evidence-strength labeling rather than through frequency counts alone (Lincoln and Guba, 1985; Nowell et al., 2017). Third, because electoral digital governance is politically sensitive, the protocol recognizes that institutional accounts are situated, partial, and shaped by access conditions (Kaiser, 2009; Aguinis and Solarino, 2019).

The design is an embedded qualitative case study. The main case is an electoral management body; the embedded units are digital arenas within that institution. This design is appropriate because digital electoral governance is inseparable from legal mandates, administrative routines, public communication, dispute channels, time pressure, and political scrutiny (Yin, 2018; James et al., 2019; Garnett and James, 2020). The embedded design allows researchers to compare how mechanisms operate across different institutional arenas without treating the organization as either a single uniform actor or a set of isolated technical platforms. The aim is analytical transferability, not statistical generalization (Yin, 2018; Lincoln and Guba, 1985).

### 3.2 Case and embedded unit selection

The case should meet four criteria: it uses digital systems in more than one electoral function; those systems face legal or public scrutiny; relevant documents or public records are available; and access to informed participants is feasible. The protocol proposes four embedded units: voter data, party and candidate verification, logistics, and results publication. These arenas cover voting rights, access to competition, operational delivery, and public explanation. Researchers may adapt the units, but each unit must have an identifiable function, digital records or systems, relevant actors, applicable rules, and plausible governance risks. A unit should be replaced, narrowed, or marked as limited-evidence if the researcher cannot identify the actors, records, documents, and risks needed to trace mechanisms.

### 3.3 Operationalization and evidence standards

Organizational capacity is defined as the observable ability of an institution to direct, coordinate, staff, secure, validate, correct, audit, explain, and learn from digital systems. Six sensitizing dimensions guide initial inquiry: strategic capacity, structural-coordinative capacity, human resource capacity, technological-data capacity, communication-legitimacy capacity, and evaluation-learning capacity. These dimensions are starting points, not fixed variables. They should be revised when evidence reveals missing practices, unexpected relationships, or context-specific mechanisms (Timmermans and Tavory, 2012).

The six dimensions are selected because they represent minimum institutional functions required to make digital systems governable in high-stakes public institutions: direction, coordination, staffing, data control, public explanation, and learning. They are derived from digital government research, data governance scholarship, organizational capacity studies, and electoral management literature (Gil-Garcia et al., 2018; Abraham et al., 2019; Garnett, 2019; Karlina et al., 2025). Each dimension becomes analytically relevant only when it can be linked to observable governance practices and supported by evidence.

The choice of six dimensions is therefore a boundary-setting decision rather than an attempt to enumerate every possible organizational attribute. Reducing the framework below these functions would risk omitting a necessary pathway of institutional governability, while expanding it too far would turn sensitizing dimensions into a descriptive inventory rather than an analytic protocol. The dimensions provide a disciplined starting structure while still allowing empirical revision through abductive analysis. In this protocol, a dimension becomes analytically relevant only when it can be linked to observable governance practices and supported by evidence. Table 2 summarizes the theoretical origins, governance functions, and illustrative mechanism questions associated with the six capacity dimensions.

**Table 2:** Theoretical derivation of capacity dimensions.

Capacity dimension	Main theoretical origin	Governance function	Illustrative mechanism question
Strategic capacity	Digital transformation, public management, organizational capacity	Direction, mandate alignment, risk prioritization	How does leadership define the purpose, authority, and limits of a digital system?
Structural-coordinative capacity	Organizational capacity, multilevel governance, electoral management	Role allocation, escalation, vertical and horizontal coordination	How are correction requests routed across units and levels?
Human resource capacity	Administrative capacity, professionalization, electoral management	Competence, training, procedural consistency	How do operators learn and apply correction, validation, and reporting routines?
Technological-data capacity	Data governance, digital government, information systems accountability	Data quality, access control, traceability, system continuity	How are records validated, changed, logged, and made auditable?
Communication-legitimacy capacity	Public accountability, electoral integrity, crisis communication	Explanation, public defensibility, correction of misunderstanding	How are digital outputs, system limits, and corrections explained to affected publics?
Evaluation-learning capacity	Organizational learning, institutional memory, policy feedback	Review, revision, documentation, learning	How does the institution revise procedures after errors, disputes, or implementation problems?

The protocol distinguishes four analytical layers: capacity, governance practice, mechanism, and governance condition. Capacity refers to an institutional ability that enables action. Governance practice refers to an observable action through which capacity is enacted. A mechanism is an explanatory pathway linking capacity, action, condition, and consequence. A governance condition is the institutional quality supported by that mechanism, such as auditability, correctability, public defensibility, accountability, or institutional learning. Table 3 defines these analytical layers and specifies their minimum evidence standards.

**Table 3:** Analytical layers and evidence standards.

Layer	Definition	Example	Minimum evidence standard
Capacity	Institutional ability that enables action	Technological-data capacity	Staff account plus rule, manual, or system-use document
Governance practice	Observable action through which capacity is enacted	Logs correction; verifies record; limits access	Interview or observation supported by procedural evidence
Mechanism	Pathway linking capacity, action, condition, and consequence	Data control enables correction traceability when authority is documented	At least two source types and rival explanations
Governance condition	Institutional quality supported by the mechanism	Auditability, public defensibility, accountability	Matrix comparison, validation feedback, or contestation record

**Figure 1:**
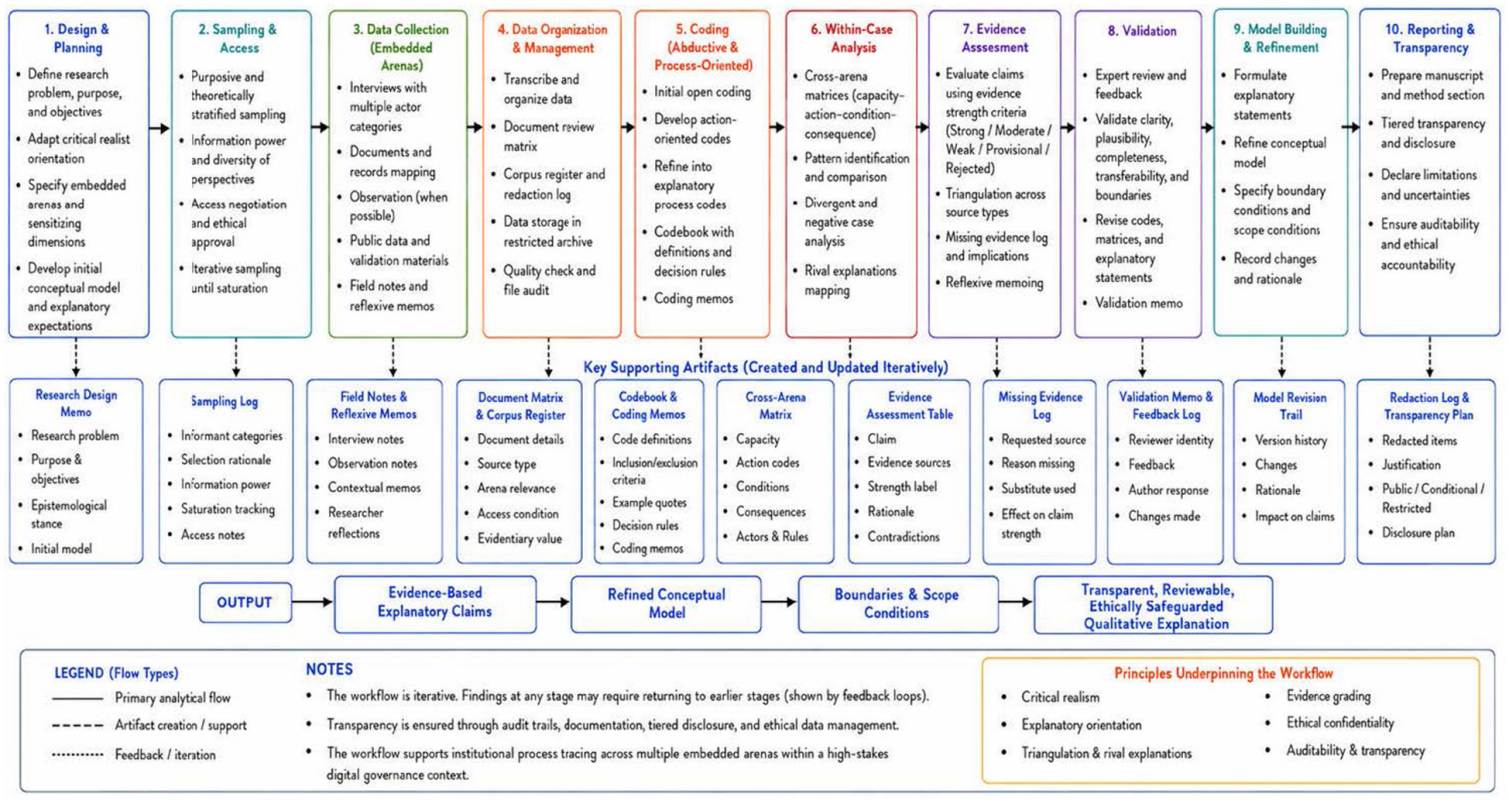
Transparent qualitative model-building workflow.

Figure 1 shows the sequence from mechanism-focused question to case selection, instrument preparation, document mapping, pilot work, fieldwork, abductive coding, mechanism coding, cross-arena comparison, pattern matching, explanation building, negative case analysis, expert validation, redaction, and refined model. Feedback arrows return from validation and negative cases to coding, matrices, and model refinement.

### 3.4 Sampling and corpus construction

Sampling is purposive, criterion-based, and functionally stratified. Participants are selected because they can explain a specific segment of the governance chain, not because they statistically represent a population (Patton, 2015; Campbell et al., 2020). The minimum sample frame should include senior decision-makers who can explain mandate interpretation, policy direction, and risk prioritization; data and technology staff who understand validation, access control, system-use routines, and audit trails; regional or local implementers who can explain vertical coordination and implementation constraints; system operators or helpdesk actors who encounter errors, correction requests, and user-facing problems; oversight, ethics, or dispute actors who observe accountability, procedural compliance, and contestation; civil society, media, or election observers who can identify public-facing problems and communication failures; electoral participants who experience procedural access, verification, correction, or challenge mechanisms; and independent experts who can assess plausibility, missing mechanisms, and boundary conditions.

Including these categories is important for reducing bias in politically sensitive electoral research. Senior officials may provide authoritative accounts but may also emphasize formal procedure or institutional success. Technical actors may explain system routines but may understate legal or public accountability issues. External observers may identify controversy but may not see internal decision processes. Electoral participants may reveal procedural access problems but may not know the full institutional chain. The protocol therefore compares claims across actor positions, documents, public records, oversight materials, and validation feedback. This source diversity reduces elite bias, technical-operator bias, official-narrative bias, and single-perspective interpretation.

A full study will usually require approximately 30 to 45 interviews, depending on access, institutional complexity, document depth, and the number of embedded units. This figure should not be treated as a fixed quota. The final sample size must be justified through information power, code saturation, and meaning saturation rather than numerical adequacy alone (Malterud et al., 2016; Hennink et al., 2017; Hennink and Kaiser, 2022). Information power is stronger when participants hold direct knowledge of a governance process, when documents are rich, and when the research question is specific to identifiable mechanisms. Code saturation may be considered reached when no new action codes, actor categories, correction routes, evidence types, or governance practices emerge after two or more consecutive interviews within an embedded arena or after the latest round of interviews across comparable arenas. Meaning saturation requires more than repeated codes; the researcher must be able to explain how a mechanism operates, under what conditions it varies, which rival explanations remain plausible, and where evidence is missing or contradictory.

For each embedded arena, researchers should seek at least three forms of evidence: one actor perspective, one legal or procedural document, and one record of implementation, public communication, oversight, or contestation. This minimum evidence triad is especially important in access-restricted institutions, where some interviews, internal documents, or operational records may be unavailable. If an arena lacks this evidentiary triad, claims for that arena should be downgraded to weak or provisional rather than presented as fully supported. Sampling decisions should be recorded in a sampling log explaining why each participant category was included, which embedded arena it informs, what analytical function it serves, what access limitations occurred, and how missing perspectives were compensated through documents, public records, observation, or expert validation.

### 3.5 Data collection and protocol procedure

The protocol proceeds in ten iterative steps. Researchers first define the mechanism-focused question and prepare a design memo. They then select the case and embedded units, prepare instruments and ethics files, map initial documents, conduct pilot interviews or pilot document coding, build the interview and document corpus, code the material, construct cross-arena matrices, build mechanism statements, and validate and refine the model. These steps are iterative rather than strictly linear, consistent with qualitative case study design, abductive analysis, and process-oriented explanation (Yin, 2018; Timmermans and Tavory, 2012; Beach and Pedersen, 2019). Evidence gathered during interviews may lead researchers back to documents; document analysis may reveal new participant categories; and negative cases may require revision of codes, matrices, or mechanism statements. Each major adjustment should be recorded in the audit trail so that the path from field evidence to model refinement remains inspectable (Nowell et al., 2017; Levitt et al., 2018).

Interview questions should be formulated from the analytical objective of tracing mechanisms rather than from broad evaluative labels. Instead of asking whether a system is transparent, accountable, effective, or trusted, the protocol requires process-oriented questions that identify actors, rules, records, decision points, correction routes, and consequences. A well-formulated question should meet four criteria: it should refer to an observable governance practice; invite the participant to reconstruct a sequence of action; ask for documentary or procedural anchors; and avoid eliciting confidential technical details, protected personal data, vulnerabilities, partisan judgments, or operational security information (Kallio et al., 2016; Patton, 2015). For example, the question “Is the system transparent?” should be replaced by “When a discrepancy is identified, who is authorized to correct it, where is the correction recorded, and how is the correction communicated to affected parties or the public?” This structure links data collection directly to mechanism building and reduces the risk that interviews produce only general opinions, institutional slogans, or unsupported evaluations (Fairfield and Charman, 2017; Ricks and Liu, 2018).

Interviews should begin with role, task, and process questions before moving to sensitive issues. Instead of asking whether a system is transparent or effective, researchers should ask how errors are detected, who authorizes correction, where correction is recorded, how disputes are escalated, how system status is explained, and how responsibility is assigned when outputs are challenged. Follow-up probes should ask participants to identify documents, decision points, actors, routines, consequences, and alternative explanations. Questions should not invite disclosure of passwords, vulnerabilities, protected data, internal security configurations, or operationally sensitive technical details because confidentiality and harm avoidance are essential in sensitive qualitative research (Kaiser, 2009; Saunders et al., 2015).

Document collection should begin before interviews and continue during fieldwork. Researchers should distinguish documents that prescribe procedure, record practice, communicate institutional claims, or register contestation. This distinction is analytically important because a legal rule, internal manual, public statement, complaint record, and oversight report do not carry the same evidentiary weight. Documents that prescribe procedure show formal expectations; documents that record practice show implementation traces; public statements show how the institution explains or defends system outputs; and contestation records show where governance claims are challenged (Bowen, 2009; Yin, 2018). Observation, when permitted, should focus on coordination, rule use, documentation, escalation, correction, and public explanation. Notes should separate observation from interpretation so that visible practice and analytical inference remain distinguishable in the audit trail (Yin, 2018; Nowell et al., 2017).

### 3.6 Analysis, mechanism building, and validation

Analysis combines abductive coding, mechanism coding, cross-arena matrices, pattern matching, explanation building, and model construction. Abductive coding begins with the six capacity dimensions and remains open to revision. Where multiple coders are involved, coding decisions, disagreements, and resolution procedures should be transparently documented (O'Connor and Joffe, 2020). Mechanism coding converts broad concepts into action-based codes, such as validates records, escalates errors, documents correction, limits access, separates unofficial from official output, explains system status, updates procedure, or routes complaint to an authorized unit. Cross-arena matrices compare actors, rules, actions, evidence, constraints, and consequences across embedded units. They should not be theme counts; their purpose is to show how governance work is performed and under what conditions it varies.

Pattern matching compares expected relationships with observed evidence (Yin, 2018). Explanation building then converts patterns into mechanism statements. Each statement should identify the capacity involved, governance practice produced, enabling and constraining conditions, evidence sources, rival explanations, evidence strength, and model implication. Process tracing is useful because it explains outcomes through linked events, decisions, and institutional actions (Fairfield and Charman, 2017; Ricks and Liu, 2018; Beach and Pedersen, 2019).

Evidence strength should be labeled as strong, moderate, weak, provisional, or rejected. To reduce subjective judgment, each label should be assigned through explicit operational criteria rather than impressionistic interpretation. The label should reflect source convergence, documentary support, actor diversity, treatment of rival explanations, unresolved contradiction, and completeness of the capacity-action-condition-consequence chain. The operational criteria and illustrative applications for each evidence-strength label are presented in Table 4.

**Table 4:** Evidence-strength decision matrix.

Evidence label	Operational criteria	Illustrative application
Strong	At least three source types converge; at least one source is legal, procedural, observational, or documentary; rival explanations have been considered; no major unresolved contradiction remains; the mechanism is supported by evidence from relevant actor categories or across comparable arenas.	A correction mechanism is supported by an official procedure, interviews with operators and oversight actors, and a public correction notice.
Moderate	At least two source types converge; minor contradictions exist but can be explained; the mechanism is plausible but not fully observed across all relevant actors or arenas.	Interview accounts and a procedural guideline support escalation practice, but no observation or implementation record is available.
Weak	Evidence rests mainly on one source type, one actor category, or retrospective accounts; documentary anchors are limited; rival explanations cannot be fully assessed.	Only officials describe a correction routine, while documents and external records are unavailable.
Provisional	The mechanism is theoretically plausible and partially indicated, but key evidence is missing, access is restricted, or the action sequence remains incomplete.	A public statement implies boundary-setting, but the internal authorization and correction route cannot be reconstructed.
Rejected	Available evidence contradicts the proposed mechanism, an alternative explanation is better supported, or a required link in the capacity-action-condition-consequence chain cannot be established.	A claimed audit trail is contradicted by oversight records showing that no reviewable record was available.

These labels are not mechanical scores. They are disciplined qualitative judgments that must be justified in a reporting memo. If a claim affects the final model, the manuscript should state which source types support it, which contradictions were considered, which rival explanations remained plausible, and why the claim was retained, downgraded, or rejected. Strong claims may inform the refined model. Moderate claims may support the model when their limits are clearly stated. Weak and provisional claims should be reported as uncertainty, not used as core evidence for the final model. Rejected claims should be documented because they show how negative evidence shaped model refinement.

Validation combines triangulation, negative case analysis, expert review, audit trails, reflexive memoing, and evidence-strength assessment. Triangulation compares interviews, documents, observation notes, public records, and validation materials to identify convergence, contradiction, and missing evidence (Denzin, 1978; Nowell et al., 2017). Negative cases prevent the model from confirming the starting framework too easily. Expert validation should test clarity, plausibility, missing mechanisms, transferability, and boundary conditions, but it should not replace field evidence. Expert feedback should therefore be treated as a validation input rather than as independent proof of a mechanism. If experts identify missing mechanisms, implausible links, or boundary conditions, these comments should be recorded in the validation memo and used to revise codes, matrices, or evidence-strength labels. Audit trails should record sampling changes, document inclusion, coding revisions, matrix decisions, validation responses, redactions, reporting choices, and changes in claim strength.

### 3.7 Managing missing evidence and bias

Missing evidence should be treated as an analytical condition, not merely as a fieldwork inconvenience. In high-stakes digital governance research, evidence may be missing because documents do not exist, access is legally restricted, officials decline interviews, records are archived in inaccessible systems, or disclosure could expose personal data, operational vulnerabilities, or politically sensitive information. The protocol therefore requires a missing-evidence log that records the requested source, expected evidentiary value, access outcome, reason for non-availability, substitute source used, and effect on claim strength. Researchers should distinguish between unavailable evidence, restricted evidence, non-existent evidence, and evidence withheld for ethical or security reasons. Each condition has different implications for inference.

Several biases are especially likely in electoral and politically sensitive digital governance research. Official actors may present *post hoc* rationalizations or minimize failure. Technical staff may overemphasize system constraints while under explaining institutional accountability. External observers may emphasize controversy more than routine practice. Documents may privilege formal procedure over actual implementation. Public statements may be designed for legitimacy rather than explanation. Researchers may also carry positional biases if they have professional, institutional, political, or prior research relationships with the organization under study. These risks should be addressed through source diversification, negative case analysis, rival-explanation memos, contradiction matrices, peer debriefing, and explicit positionality statements.

Bias mitigation should be visible in the audit trail. For each candidate mechanism, the researcher should ask: which actors support this claim, which actors might contest it, what documents anchor it, what evidence is missing, what rival explanation remains plausible, and what would cause the claim to be downgraded or rejected? If a mechanism is supported only by elite interviews, it should not be labeled strong unless documentary or external evidence confirms the relevant action sequence. If evidence is uneven across embedded arenas, the model should show that unevenness rather than presenting all pathways as equally supported.

### 3.8 Tiered transparency and confidentiality

Transparency in sensitive digital governance research cannot mean unrestricted disclosure of all raw materials. Election-related digital systems may involve personal data, operational security, internal access procedures, contested political events, and identifiable officials. Full disclosure may expose participants or institutions to avoidable harm. At the same time, confidentiality cannot be used to shield weak inference. The protocol therefore uses a tiered transparency framework that separates public artifacts, conditional artifacts, restricted artifacts, and non-disclosable materials. Table 5 summarizes the transparency tiers, their access conditions, and the corresponding reviewer-verification routes.

**Table 5:** Tiered transparency framework.

Transparency tier	Artifact type	Access condition	Reviewer verification route
Public	Published laws, regulations, public guidelines, public statements, public correction notices, published oversight documents	Publicly shareable	Direct citation, appendix listing, public document matrix
Conditional	De-identified codebook, aggregated interview matrix, redacted document matrix, summarized validation memo	Shareable with redaction or ethics approval	Supplementary file, anonymized excerpt, structured summary
Restricted	Full corpus register, full interview transcripts, internal memos, detailed redaction logs, sensitive validation records	Available only to authorized research team or ethics-approved reviewers	Audit-trail summary, independent ethics confirmation, confidential reviewer access where permitted
Non-disclosable	Passwords, protected personal data, system vulnerabilities, security configurations, identifiable sensitive testimony	Not shareable	Exclusion statement, redaction rationale, confirmation that no claim relies on unsafe disclosure

Reviewers can assess restricted claims through procedural transparency even when raw materials are unavailable. The manuscript or supplement should specify the source type supporting each major claim, the number and category of sources, the relevant embedded arena, the evidence-strength label, and the redaction rationale. Where possible, authors should provide anonymized excerpts, paraphrased evidence summaries, document metadata, or public documentary anchors. If none of these can be safely shared, the claim should be downgraded unless it is supported by other shareable evidence. This approach balances reviewability with confidentiality, security, and legal obligations.

### 3.9 Researcher positionality

Researcher positionality is a methodological and ethical issue in electoral research, not only a matter of personal reflection. Access to electoral institutions may be mediated by formal authority, prior professional networks, academic affiliation, political sensitivity, or gatekeeper approval. These conditions can influence who agrees to participate, what participants disclose, how documents are made available, and how researchers interpret contested events. The protocol therefore requires a positionality memo before fieldwork and updated reflexive entries during data collection and analysis.

The positionality memo should document relevant institutional affiliations, prior assumptions, access pathways, gatekeeper relationships, possible dependencies, and anticipated risks of overidentification with either institutional or external critics. During analysis, the researcher should record moments when positionality may have shaped sampling, questioning, interpretation, redaction, or claim strength. Where access is mediated by one office or actor, researchers should actively seek counter-perspectives from other units, oversight actors, public documents, affected participants, media records, or independent experts. Reflexivity is therefore linked directly to evidence assessment: positional risks should affect how claims are labeled, bounded, validated, and reported.

## 4 Anticipated results

### 4.1 Expected form of results

Applying this protocol should produce a traceable qualitative model, not a statistical estimate, technical certification, cybersecurity audit, or population-level finding. The anticipated results should therefore be judged by procedural clarity, evidentiary grounding, analytical transparency, ethical care, and usefulness for adaptation. The central product is a model explaining how organizational capacity becomes digital governance practice across embedded arenas. A strong results section will not present a polished diagram alone. It will show how evidence moved from interviews, documents, observation notes, public records, validation feedback, and redaction decisions into mechanism-based claims. Readers should be able to see what was included, how it was coded, where sources converged, which rival explanations remained plausible, which claims were rejected, and how the final model changed during analysis. This form of reporting is especially important when raw materials cannot be fully shared because of confidentiality, political sensitivity, or security risk (Nowell et al., 2017; Levitt et al., 2018).

### 4.2 Analytical outputs

Protocol implementation is expected to generate ten analytical outputs: a corpus register, document review matrix, preliminary codebook, mechanism-oriented codebook, cross-arena matrix, mechanism map, validation memo, redaction log, reporting memo, and refined model. These outputs make the evidentiary base, coding process, comparison logic, validation procedure, redaction decisions, and final mechanism claims reviewable. Table 6 summarizes each output, its quality function, and recommended reporting location.

**Table 6:** Anticipated outputs and quality functions.

Output	Main content	Quality function	Recommended location
Corpus register	Interviews, documents, observations, validation records, memos	Establishes the evidentiary base	Supplement or restricted archive
Document review matrix	Source, date, legal status, arena, access, value	Makes documentary evidence traceable	Supplement, with redactions
Preliminary codebook	Deductive and emergent codes	Shows conceptual application and revision	Supplement
Mechanism codebook	Action codes, conditions, consequences	Prevents purely thematic reporting	**Supplement**
Cross-arena matrix	Comparison across embedded units	Supports within-case comparison	Article table or **supplement**
Mechanism map	Capacity-to-practice pathways	Displays explanatory structure	Main article figure
Validation memo	Expert feedback and unresolved issues	Tests plausibility and transferability	Summarized in text
Redaction log	Removed details and rationale	Balances transparency with safety	Restricted archive or summary
Reporting memo	Evidence limits and claim strength	Reduces overclaiming	**Supplement**
Refined model	Revised capacity-to-governance explanation	Provides final analytical output	Main article figure and narrative

### 4.3 Refined model structure

The refined model should treat capacity as a set of institutional abilities enacted through governance mechanisms, not as an abstract organizational attribute. Strategic, structural-coordinative, human resource, technological-data, communication-legitimacy, and evaluation-learning capacities should be linked to observable practices such as mandate alignment, role allocation, validation, correction, public explanation, and procedural revision. These links are propositions to be tested, revised, weakened, or rejected through evidence rather than findings assumed in advance.

**Figure 2:**
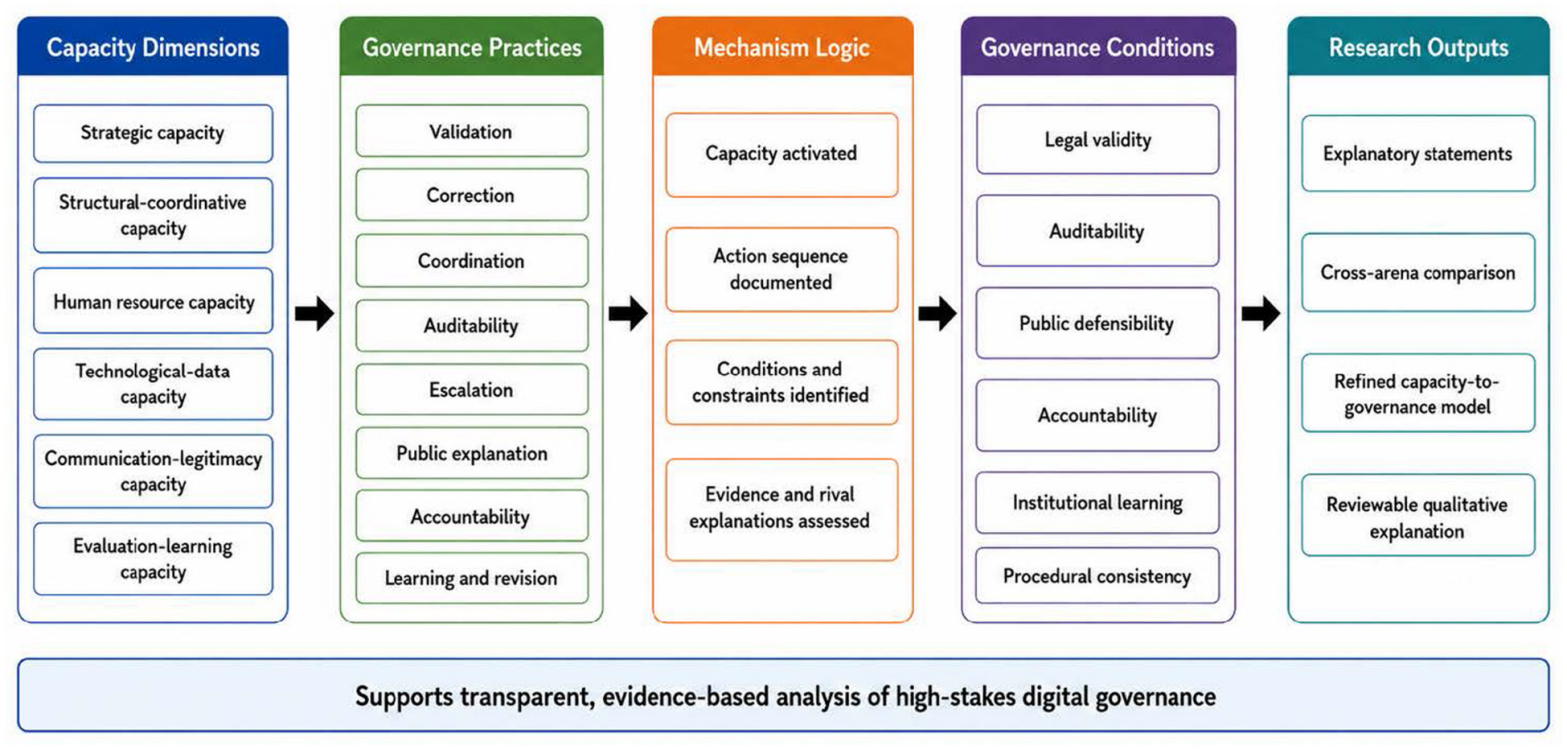
Analytical architecture for capacity-to-governance mechanism tracing.

Figure 2 presents four analytical layers–capacity dimensions, governance practices, mechanism logic, and governance conditions-leading to the research outputs. Capacity dimensions include strategic, structural-coordinative, human resource, technological-data, communication-legitimacy, and evaluation-learning capacity. Governance practices include validation, correction, coordination, auditability, escalation, explanation, accountability, and learning. Mechanism statements link capacity, action sequence, condition, evidence, and rival explanation. Governance conditions include legal validity, auditability, public defensibility, accountability, and institutional learning. Dashed arrows show that relationships are tested and revised through evidence, negative cases, validation, and redaction review.

### 4.4 Brief pilot empirical vignette: results publication and public defensibility

To demonstrate bounded methodological feasibility without presenting a full institutional evaluation, this pilot vignette applies selected protocol steps to publicly available materials concerning SIREKAP in Indonesia's 2024 electoral administration. The public corpus included Komisi Pemilihan Umum Decision No. 66 of 2024 concerning the technical procedures for voting and vote counting, Komisi Pemilihan Umum Decision No. 219 of 2024 concerning the implementation of vote-recapitulation procedures, and publicly available institutional explanations concerning the function and legal status of digitally displayed electoral information (Komisi Pemilihan Umum Republik Indonesia, 2024a,b). These sources were selected because they allow a limited reconstruction of how an electoral management body distinguishes digital publication from legally authoritative electoral documentation. The vignette does not evaluate SIREKAP's technical reliability, cybersecurity, accuracy rate, or overall institutional performance. Its purpose is solely to demonstrate how the protocol transforms documentary materials into a bounded and evidence-graded mechanism claim. Table 7 summarizes this pilot evidence-to-mechanism transformation.

**Table 7:** Pilot evidence-to-mechanism pathway.

Analytical step	Pilot material	Transformation
Public source mapping	Technical or regulatory documents defining system function	Classified as legal/procedural evidence
Public communication mapping	Institutional explanation of digital output status	Coded for explanation and boundary-setting
Contested setting mapping	Public controversy, oversight statement, or media record	Coded for public contestation and legitimacy pressure
Initial broad code	Transparency; results publication	Replaced because it is too broad
Action-based code	Distinguishes preliminary from authoritative record; explains legal status; routes discrepancy	Mechanism-oriented coding
Matrix entry	Results publication: actors, rules, action, evidence, contestation	Cross-arena comparison
Mechanism statement	Communication-legitimacy capacity supports public defensibility through documented boundary-setting and correction explanation	Candidate mechanism
Evidence rating	Moderate or provisional	Claim-strength label reflecting public-only corpus

The pilot began with the mechanism-focused question: how can an electoral management body maintain public defensibility when a digital results-publication tool displays information before formal legal recapitulation is completed? Each document was entered into a document-review matrix containing its issuer, date, formal status, intended function, embedded arena, governance relevance, access condition, and evidentiary value. The analysis distinguished three documentary functions: sources prescribing voting, counting, or recapitulation procedures; sources defining or explaining the status of digital information; and publicly available materials indicating that discrepancies or interpretations of system outputs had become contested. This distinction prevented regulatory provisions, institutional explanations, and contestation records from being treated as evidentially equivalent.

Initial coding generated broad concepts such as transparency, public trust, results publication, and system accuracy. Because these labels did not identify an institutional action sequence, they were replaced with mechanism-oriented action codes: defines the function of the digital tool; distinguishes displayed information from the authoritative legal record; preserves the formal recapitulation channel; explains the status and limits of digital output; identifies or clarifies a discrepancy; and directs challenges toward an authorized correction or recapitulation procedure. The codes were entered into the results-publication arena of the cross-arena matrix and connected to the relevant actors, rules, documentary evidence, public explanation, contestation, and missing evidence. Because the pilot relied on public documents and did not include interviews, internal authorization records, system logs, or direct observation, the resulting claim could not be classified as strong.

The pilot produced the following candidate mechanism: communication-legitimacy capacity supports public defensibility when an electoral management body explicitly distinguishes preliminary digital publication from legally authoritative recapitulation, identifies the applicable decision channel, and provides an institutionally authorized route for clarification or correction. The claim was rated moderate where at least two independent public source types supported the boundary between digital display and formal recapitulation, and provisional where the internal authorization or correction sequence could not be reconstructed. This mechanism statement is deliberately bounded. It does not demonstrate that all discrepancies were corrected, that the digital system was technically reliable, or that public trust increased. Instead, it demonstrates the protocol's practical sequence from corpus selection, document classification, broad coding, action coding, matrix construction, evidence assessment, and missing-evidence identification to a qualified mechanism statement that remains open to revision through interviews, observation, oversight evidence, and expert validation.

### 4.5 Advantages of protocol application

The main advantage of protocol application is reviewability. By requiring a documented chain from corpus construction to coding, matrix comparison, validation, redaction, and model refinement, the protocol reduces the gap between qualitative evidence and final explanatory claims. Cross-arena matrices also prevent the analysis from being confined to one platform or organizational unit. At the same time, the protocol supports safe reporting by allowing researchers to describe governance mechanisms without disclosing passwords, protected personal data, system architecture, security configurations, or operational vulnerabilities (Kaiser, 2009; Saunders et al., 2015).

### 4.6 Limitations and boundary conditions

The anticipated results will remain bounded by access, evidence quality, and institutional sensitivity. The protocol cannot estimate causal effects, measure public trust, certify cyber security, evaluate software performance, or determine whether a system is technically secure. Findings should be presented as mechanism-based explanations grounded in a specific institutional setting. Transferability can be claimed only when researchers specify the conditions that make comparison plausible: legal mandate, digital maturity, data governance arrangements, electoral cycle stage, oversight environment, public contestation, and evidence availability.

Uneven evidence across embedded units will affect model strength. If results publication has richer documents than logistics, claims about results publication may be stronger. Limited access to officials may weaken reconstruction of decision authority. Defensive interviews may understate conflict or uncertainty. Missing documents may narrow claims about correction or auditability. These limits should appear in the results narrative, the reporting memo, and the refined model. A credible application of the protocol should state where evidence is strong, where it is thin, which mechanisms were rejected, and which remain provisional.

### 4.7 Transferability assessment checklist

Transferability should be assessed before applying the protocol to courts, welfare agencies, tax authorities, population registries, public health systems, anti-corruption bodies, or other high-stakes public institutions. The protocol is transferable only when the target institution shares enough governance conditions with the original setting to make mechanism tracing meaningful. Table 8 presents the criteria and implications used to assess the protocol's transferability.

**Table 8:** Transferability assessment checklist.

Assessment criterion	Guiding question	Transferability implication
Legal mandate	Does the institution produce decisions or records with legal or rights-related consequences?	High relevance if digital outputs affect rights, eligibility, obligation, or accountability
Digital dependence	Are core procedures mediated by digital records, platforms, or public information systems?	High relevance when digital systems structure institutional action
Auditability requirement	Must decisions be reviewed, challenged, corrected, or explained?	High relevance when records must remain traceable
Public scrutiny	Are outputs politically, socially, legally, or reputationally contested?	High relevance when public defensibility matters
Data sensitivity	Does the system process personal, confidential, or security-sensitive data?	Requires tiered transparency and redaction
Organizational complexity	Are multiple units, levels, or external actors involved?	Supports embedded case design
Evidence availability	Are documents, interviews, public records, or observations accessible?	Determines feasible claim strength
Correction mechanism	Are there procedures for error detection, correction, escalation, or appeal?	Enables mechanism tracing
Boundary clarity	Is the legal status of digital outputs clear or contested?	Important for studying public defensibility
Ethical risk	Could disclosure harm participants, rights holders, or institutional security?	Requires restricted archive and safe reporting

## 5 Discussion

### 5.1 Methodological contribution

The methodological innovation of this protocol does not reside in claiming that interviews, document analysis, case studies, abductive coding, or process tracing are individually new. Its contribution lies in integrating these established procedures into a single inspectable architecture for mechanism-oriented qualitative model-building. Conventional qualitative studies may provide rich descriptions and theoretically meaningful categories, but the intermediate analytical decisions connecting corpus selection, coding, within-case comparison, rival explanations, evidence strength, redaction, validation, and final model construction are not always reported systematically. The proposed protocol makes those intermediate decisions part of the method itself by requiring a corpus register, sampling log, document matrix, action-oriented codebook, cross-arena matrix, mechanism statement, missing-evidence log, validation memo, evidence-strength assessment, redaction record, and model-revision trail. The added value is therefore procedural integration and reviewability rather than the invention of an entirely new qualitative technique. Table 9 summarizes the protocol's contributions to qualitative methods, research transparency, digital governance, data governance, and electoral integrity.

**Table 9:** Discussion-level contributions of the protocol.

Contribution area	Problem addressed	Added value of the protocol
Qualitative methods	Final models often obscure inferential steps	Makes movement from evidence to mechanism inspectable
Research transparency	Raw data may be unsafe to share	Uses artifacts, logs, and memos for procedural transparency
Digital governance	Systems are often assessed as tools	Studies institutional governability and accountability work
Data governance	Data rules may be separated from practice	Traces validation, correction, access, audit, and explanation
Electoral integrity	Technology risks become political disputes	Links capacity to public defensibility and institutional credibility

Compared with a conventional embedded case study, the protocol provides more explicit rules for translating evidence from organizational subunits into graded mechanism claims. Compared with process tracing, it gives greater attention to corpus construction, qualitative coding, differences between formal, reported, visible, and contested practice, and the ethical problem of verifying claims when supporting materials cannot be disclosed publicly. Compared with thematic analysis, it requires researchers to move beyond recurrent topics toward action sequences connecting capacity, practice, condition, and consequence. Its embedded arenas also prevent the electoral management body from being treated either as a uniform organizational actor or as a collection of disconnected technologies. The protocol thus combines contextual depth, mechanism reconstruction, within-case comparison, explicit uncertainty, and tiered transparency in a form designed for legally sensitive, politically contested, and data-intensive institutions.

### 5.2 Relationship to grounded theory, institutional ethnography, and critical discourse analysis

The protocol builds on but differs from three established methodological traditions. Grounded theory is relevant because it provides systematic procedures for moving from data to conceptual categories and theory construction (Strauss and Corbin, 1998; Charmaz, 2014). However, the present protocol does not aim to generate theory primarily through progressive coding from empirical data. It begins with sensitizing dimensions derived from digital governance, organizational capacity, data governance, and electoral management literature, then uses abductive analysis to revise those dimensions. The objective is not category saturation alone, but evidence-graded mechanism statements that link capacity, action, condition, and consequence.

Institutional ethnography is also relevant because it studies how everyday work is coordinated through texts, rules, and institutional relations (Smith, 2005). The protocol shares its concern with institutional work, documentary mediation, and administrative routines. However, it does not adopt a full ethnographic standpoint approach or require prolonged immersion in everyday work processes. Instead, it offers a practical embedded case protocol for contexts where access may be restricted, observation may be limited, and public documents, interviews, and validation materials must be combined carefully.

Critical discourse analysis contributes to understanding how institutional language, public communication, and legitimacy claims are constructed (Fairclough, 1995, 2003). This protocol uses public statements and institutional texts as evidence, but it does not treat discourse as the primary object of explanation. Its focus is broader: how communication interacts with legal authority, data validation, correction routines, audit trails, and accountability mechanisms. The protocol therefore complements discourse analysis by linking textual claims to institutional action sequences and evidence-strength assessment.

### 5.3 From themes to mechanisms

A central methodological claim of the article is that themes should not be mistaken for explanations. Terms such as transparency, accountability, data quality, trust, and auditability are useful sensitizing concepts, but they remain analytically thin unless the researcher can show what actors did, under which rules, with which records, and with what consequences. The protocol therefore treats broad themes as provisional and requires researchers to translate them into mechanism statements. This approach draws on abductive analysis and process-oriented qualitative explanation, which link events, actions, and conditions rather than merely naming categories (Timmermans and Tavory, 2012; Fairfield and Charman, 2017; Ricks and Liu, 2018; Beach and Pedersen, 2019).

For example, auditability is not a mechanism by itself. It becomes a mechanism only when a study can show how an institution records a decision, preserves the relevant evidence, assigns authority for correction, and makes the record available for review. The same is true for public explanation. A public notice does not automatically demonstrate transparency; it may clarify responsibility, obscure a procedural gap, or redirect attention away from contested evidence. Action codes, cross-arena matrices, rival explanations, evidence-strength labels, and validation memos help prevent a conceptual model from becoming a diagram of attractive terms rather than a grounded explanation of institutional practice (Nowell et al., 2017; Bingham, 2023).

### 5.4 Transparency without unsafe disclosure

The protocol also clarifies what transparency can reasonably mean in politically sensitive digital governance research. Full openness is not always ethical, lawful, or safe. Studies of electoral systems may involve officials, operators, protected records, cybersecurity concerns, disputed events, and reputational risk. In such settings, responsible transparency cannot mean publishing everything. It means providing enough information for readers to understand how claims were produced while protecting participants and avoiding operational harm. This position is consistent with qualitative confidentiality research, which shows that anonymization, aggregation, and selective reporting shape both ethics and evidence quality (Kaiser, 2009; Saunders et al., 2015).

This is why the protocol emphasizes tiered artifact-based transparency. Rather than requiring full disclosure of raw data, it uses corpus registers, document matrices, codebooks, mechanism statements, validation memos, redaction logs, evidence-strength tables, and audit trails to make inference reviewable. These artifacts help distinguish unavailable, restricted, and non-existent evidence, while requiring researchers to state how each condition affects claim strength (Levitt et al., 2018; Aguinis and Solarino, 2019).

### 5.5 Implications for digital governance research

The protocol shifts attention from digital adoption to institutional governability. A digital system is governable when an organization can define its purpose, assign responsibility, control access, validate records, correct errors, document decisions, manage risk, explain outputs, and revise procedures after implementation. This matters because public-sector digital transformation is often discussed through integration, innovation, data use, service maturity, or organizational performance (Gil-Garcia et al., 2018; Mergel et al., 2019; Vial, 2019). Those perspectives are important, but they can miss the institutional labor required to make digital outputs defensible when they are questioned.

Electoral administration makes this issue especially visible. Voter lists, nomination files, logistics records, and results information shape rights, competition, operational readiness, public knowledge, and confidence in outcomes. A technical failure, ambiguous output, or delayed correction can quickly become a legitimacy problem. The protocol therefore treats data governance as practice rather than as policy text or technical architecture alone. Data governance scholarship emphasizes decision rights, standards, access rules, quality control, stewardship, and accountability (Khatri and Brown, 2010; Abraham et al., 2019; van Donge et al., 2022). The protocol translates those concerns into qualitative questions: who validates data, who can change records, how corrections are documented, how legal status is communicated, and how accountability is maintained when digital outputs become contested.

### 5.6 Public defensibility and electoral integrity

Public defensibility is useful for studying digital electoral governance because it captures what institutions must do when digital outputs become consequential. It refers to an institution's ability to explain and justify a digital system's status, limits, outputs, and correction procedures in ways consistent with legal authority and public accountability. It does not require full technical disclosure. In sensitive systems, excessive disclosure may expose vulnerabilities or endanger participants. What matters is whether the institution provides enough explanation, documentation, and correction capacity for the public, oversight actors, and affected parties to interpret digital outputs responsibly.

This concept links the protocol to electoral integrity without turning the article into a substantive theory of elections. Electoral integrity research shows that credible elections depend on law, administration, impartiality, transparency, dispute resolution, and public trust (Birch, 2011; Norris, 2014; James et al., 2019). Electoral management research further shows that capacity and implementation shape whether procedures are consistent, credible, and accepted (Garnett, 2019; Garnett and James, 2020). Digital technologies may improve speed, access, documentation, and public information, but they may also intensify risks related to data error, system failure, cybersecurity, vendor dependence, misinformation, and contested interpretation (Alvarez et al., 2009; Haque and Carroll, 2020; Krimmer et al., 2021). The protocol helps researchers study those risks as governance problems rather than as purely technical failures.

### 5.7 Boundary conditions and future use

The protocol has clear boundaries. It is not designed to certify software, audit cybersecurity, estimate causal effects, measure public trust, or evaluate technical performance. Its value lies in tracing institutional mechanisms where digital systems carry legal authority, public accountability, or legitimacy claims. It will be less useful for routine administrative systems with limited public consequence or for research questions that require direct measurement of system performance.

Future applications can extend the protocol in three directions. Comparative studies can apply it across electoral management bodies to examine how mechanisms vary by legal framework, administrative structure, digital maturity, or political context. Multimethod studies can combine protocol outputs with surveys, system logs, complaint data, media analysis, audit findings, or public communication records. Researchers can also adapt the design to courts, welfare systems, digital identity agencies, population registries, tax agencies, public health systems, and anti-corruption bodies, but only after applying the transferability checklist. In all cases, the central question remains the same: how do institutions make digital systems governable, accountable, and publicly defensible under scrutiny?

## 6 Conclusion

Digital governance research often explains systems through performance, adoption, maturity, usability, security, or public confidence. These perspectives remain useful, but they leave a crucial institutional question only partly answered: how do organizations make digital systems governable once their outputs affect rights, procedures, accountability, and public legitimacy? The qualitative methods protocol developed in this article addresses that question by offering a structured way to trace validation, correction, coordination, auditability, explanation, accountability, and learning as observable institutional work.

The main contribution is methodological. Embedded qualitative case design allows researchers to compare arenas within a single institution without treating the organization as either a uniform actor or a collection of isolated platforms. Voter data, party and candidate verification, logistics, and results publication become sites for examining how actors, rules, records, routines, corrections, explanations, and oversight relations interact. Such comparison makes qualitative model-building more disciplined because broad concepts such as transparency, accountability, capacity, and auditability gain explanatory value only when connected to documented actions, evidence chains, rival explanations, and mechanism statements.

A second contribution concerns safe research transparency. Sensitive institutional research cannot always disclose raw material, especially when election technology, public-sector data systems, cyber security concerns, or politically contested records are involved. Yet protection should not justify opaque inference. The protocol therefore promotes procedural transparency through corpus registers, document matrices, codebooks, cross-arena matrices, validation memos, evidence-strength labels, redaction logs, transferability checklists, and audit trails.

The protocol is not a substitute for software certification, cyber security auditing, causal estimation, usability testing, or population-level trust measurement. Its purpose is to make institutional mechanisms visible, assessable, and transferable under clearly stated boundary conditions. Used with methodological discipline and ethical restraint, the protocol can strengthen qualitative research on digital governance by making the path from evidence to explanation more transparent, accountable, and reviewable.

## Data Availability

The original contributions presented in the study are included in the article/Supplementary material, further inquiries can be directed to the corresponding author.
