# Supplementary material for: Transparent qualitative model-building in high-stakes digital governance: a methods protocol for tracing institutional mechanisms
Source: Front Res Metr Anal. 2026 Jul 27;11:1889262. doi: 10.3389/frma.2026.1889262 (PMC13454106; doi:10.3389/frma.2026.1889262)
Supplement: Supplementary file 1 [file Data_Sheet_1.docx]

Supplementary Material

# 1 Supplementary Figures and Tables

## 1.1 Supplementary Figures

**Supplementary Figure 1.** The workflow shows how the protocol moves from a mechanism-focused research question to embedded case design, corpus construction, mechanism-oriented analysis, validation, redaction, and model refinement. The figure is procedural rather than empirical; it represents the recommended analytical sequence for applying the protocol.

## 1.2 Supplementary Figures

**Supplementary Figure 2.** The figure presents the analytical logic of the protocol. It does not claim that every capacity dimension automatically produces every governance condition. Relationships among capacities, practices, mechanisms, and conditions must be tested through evidence, negative cases, rival explanations, and validation. The architecture is intended to guide mechanism-oriented qualitative analysis rather than to predetermine findings.

## 1.3 Supplementary Tables

## Supplementary Tables 1. Materials, Instruments, and Methodological Functions

| Category | Required item | Methodological function |
| --- | --- | --- |
| Data materials | Interview transcripts | Reconstruct routines, decisions, constraints, interpretations, and action sequences |
| Data materials | Legal and procedural documents | Establish mandates, authority, legal status, correction pathways, and boundaries |
| Data materials | Public statements, oversight records, and observation notes | Trace explanation, contestation, institutional response, and visible practice |
| Data materials | Validation records | Document expert feedback, uncertainty, model revision, and boundary conditions |
| Instruments | Interview protocol and document matrix | Standardize questioning and classify documentary evidence |
| Instruments | Observation and coding guides | Keep observation safe and support consistent coding and comparison |
| Instruments | Cross-arena matrix and validation form | Compare mechanisms and test plausibility, transferability, and limits |
| Analytical tools | QDA software, spreadsheets, secure storage, version log, and redaction template | Support coding, retrieval, matrices, data protection, audit trails, and safe reporting |

*Note.* QDA = qualitative data analysis. The table identifies the minimum materials and tools required to implement the protocol. Items may be adapted to institutional context, access conditions, and ethics requirements, but each study should preserve the four core functions: corpus construction, analytic traceability, participant protection, and safe reporting.

## Supplementary Tables 2. Analytical layers and evidence standards

| Layer | Definition | Example | Minimum evidence standard |
| --- | --- | --- | --- |
| Capacity | Institutional ability that enables action | Technological-data capacity | Staff account plus rule, manual, or system-use document |
| Governance practice | Observable action through which capacity is enacted | Logs correction; verifies record; limits access | Interview or observation supported by procedural evidence |
| Mechanism | Pathway linking capacity, action, condition, and consequence | Data control enables correction traceability when authority is documented | At least two source types and rival explanations |
| Governance condition | Institutional quality supported by the mechanism | Auditability, public defensibility, accountability | Matrix comparison, validation feedback, or contestation record |

*Note.* The table distinguishes concepts that are often conflated in qualitative model-building. Capacity refers to an institutional ability, governance practice to observable action, mechanism to an explanatory pathway, and governance condition to the institutional quality supported by the pathway. The evidence standards are minimum expectations; stronger claims require convergence across multiple sources and attention to negative cases.

## Supplementary Tables 3. Anticipated outputs and quality functions

| Output | Main content | Quality function | Recommended location |
| --- | --- | --- | --- |
| Corpus register | Interviews, documents, observations, validation records, memos | Establishes the evidentiary base | Supplement or restricted archive |
| Document review matrix | Source, date, legal status, arena, access, value | Makes documentary evidence traceable | Supplement, with redactions |
| Preliminary codebook | Deductive and emergent codes | Shows conceptual application and revision | Supplement |
| Mechanism codebook | Action codes, conditions, consequences | Prevents purely thematic reporting | Supplement |
| Cross-arena matrix | Comparison across embedded units | Supports within-case comparison | Article table or supplement |
| Mechanism map | Capacity-to-practice pathways | Displays explanatory structure | Main article figure |
| Validation memo | Expert feedback and unresolved issues | Tests plausibility and transferability | Summarized in text |
| Redaction log | Removed details and rationale | Balances transparency with safety | Restricted archive or summary |
| Reporting memo | Evidence limits and claim strength | Reduces overclaiming | Supplement |
| Refined model | Revised capacity-to-governance explanation | Provides final analytical output | Main article figure and narrative |

*Note.* The anticipated outputs are research artifacts rather than empirical findings. Their purpose is to make qualitative inference inspectable when raw data cannot be fully shared because of confidentiality, political sensitivity, legal restriction, or security risk.

## Supplementary Tables 4. Illustrative evidence-to-mechanism pathway

| Analytical step | Illustrative material | Transformation |
| --- | --- | --- |
| Source material | Guideline on preliminary results | Classified as legal/procedural evidence |
| Source material | Interview with communication staff | Coded for explanation practice |
| Source material | Public correction notice | Coded for boundary-setting and correction |
| Initial code | Transparency | Replaced because it is too broad |
| Action-based code | Explains system status; separates preliminary from official output | Mechanism-oriented code |
| Matrix entry | Results publication: actors, rule, action, evidence, consequence | Cross-arena comparison |
| Mechanism statement | Communication-legitimacy capacity supports public defensibility through documented explanation of legal status and correction routes | Candidate mechanism |
| Evidence rating | Strong, moderate, weak, or provisional | Claim-strength label |

*Note.* The example is illustrative and does not report empirical findings. It shows how a broad theme, such as transparency, can be converted into a mechanism statement only after the researcher identifies actions, evidence sources, conditions, consequences, and rival explanations.

## Supplementary Tables 5. Discussion-Level Contributions of the Protocol

| Contribution area | Problem addressed | Added value of the protocol |
| --- | --- | --- |
| Qualitative methods | Final models often obscure inferential steps | Makes movement from evidence to mechanism inspectable |
| Research transparency | Raw data may be unsafe to share | Uses artifacts, logs, and memos for procedural transparency |
| Digital governance | Systems are often assessed as tools | Studies institutional governability and accountability work |
| Data governance | Data rules may be separated from practice | Traces validation, correction, access, audit, and explanation |
| Electoral integrity | Technology risks become political disputes | Links capacity to public defensibility and institutional credibility |

*Note.* The table summarizes the article’s discussion-level contribution across methodological and substantive fields. It should appear in the Discussion section after the subsection on safe transparency or before the subsection on implications for digital governance research.
